# Semi-mechanistic population pharmacokinetic model incorporating glutathione S-transferase activity for personalized busulfan dosing in pediatric allogeneic hematopoietic cell transplantation

**DOI:** 10.3389/fphar.2025.1632588

**Published:** 2025-08-29

**Authors:** Di Cao, Xiaowen Qian, Ping Wang, Xinyi Zheng, Shan Huang, Zhonglin Wei, Wenjin Jiang, Ling Yu, Xin Jiang, Ying Yu, Junjun Mao, Xiaowen Zhai

**Affiliations:** ^1^ Medical Affairs Office, National Children’s Medical Center, Children’s Hospital of Fudan University, Shanghai, China; ^2^ Department of Hematology, National Children’s Medical Center, Children’s Hospital of Fudan University, Shanghai, China; ^3^ Department of Pharmacy, Huashan Hospital of Fudan University, Shanghai, China; ^4^ Department of Pediatrics, Tongji Hospital, Tongji Medical College, Huazhong University of Science and Technology, Wuhan, China; ^5^ Department of Hematology and Oncology, Children’s Hospital of Soochow University, Suzhou, China

**Keywords:** busulfan, population pharmacokinetics, glutathione S-transferase activity, precision dosing, pediatric transplantation, virtual clinical trial

## Abstract

**Background:**

Busulfan is known for its high inter- and intra-individual pharmacokinetics/pharmacodynamics (PK/PD) variability, especially in children. Therefore, we aimed to identify factors affecting PK variability of busulfan in pediatric allogeneic hematopoietic cell transplantation (HCT) recipients and investigate the effect of glutathione S-transferase (GST) activity on busulfan metabolism using a semi-mechanistic population PK model.

**Methods:**

Overall, 636 whole-blood busulfan concentrations from 65 pediatric HCT recipients were analyzed using nonlinear mixed-effects modeling. A semi-mechanistic population PK model was developed to describe busulfan metabolism in response to glutathione (GSH) depletion. The effects of potential covariates were selected based on previous study and physiologically-based theoretical mechanisms. Virtual clinical trials were conducted to compare different dosing strategies, and model-based optimal dosing regimen was recommended.

**Results:**

A two-compartment model with first-order absorption was selected to describe busulfan PK. A GSH compartment was added to represent the relative amount of GSH available at any time. The estimated mean clearance of busulfan was 9.57 L h^−1^ (relative standard error: 10.8%). Busulfan disposition was best described by including normal fat mass (NFM) allometrically and GST enzyme activity on S_GSH_ exponentially. The S_GSH_ increased by 40.6% as GST enzyme activity increased from 0.9 nmol/min/mL to 20.7 nmol/min/mL. Patients with weights (WT) of 9–16 kg are at high risk of sinusoidal obstructive syndrome (SOS) when receiving WT-based dosing strategy.

**Conclusion:**

NFM, age-dependent maturation function, and GST enzyme activity may contribute to busulfan PK variability. The WT-based dosing strategy showed a higher risk of SOS than the age-based dosing strategy in 9–16 kg patients.

## 1 Introduction

Busulfan, a bifunctional DNA-alkylating agent, is widely applied as a chemotherapeutic in combination with cyclophosphamide, cytarabine, and fludarabine before allogeneic hematopoietic cell transplantation (HCT) (Chen et al., 2018; Lawson et al., 2021). This treatment can reduce the immune response to avoid graft rejection and provide favorable conditions for donor cell engraftment. Critically, subtherapeutic drug exposure levels correlate with increased relapse rates or graft failure, while supratherapeutic concentrations are linked to a greater risk of severe toxicities and treatment-related mortality (Bartelink et al., 2016).

The clinical application of busulfan is complicated by its high inter- and intra-individual pharmacokinetics/pharmacodynamics (PK/PD) variability, particularly in children (Lawson et al., 2021). Consequently, intravenous (IV) administration is preferred in children because of the higher bioavailability and reduced PK variability compared to oral formulations (Palmer et al., 2016). Following IV infusion, busulfan undergoes rapid distribution and binds extensively to erythrocytes (approximately 47%) and plasma proteins (approximately 32%). Hepatic metabolism occurs primarily through conjugation with glutathione (GSH) mainly through glutathione S-transferases (GSTs) (Lawson et al., 2021; Scian et al., 2016), with renal excretion playing a minor role, only about 2% of busulfan is detected unmetabolized in the urine (Hassan et al., 1989).

Furthermore, owing to its narrow therapeutic index and large PK/PD variability, administering an initial busulfan IV dose based only on body weight (WT) may result in failure to reach the target therapeutic window (Ben Hassine et al., 2021; Huang et al., 2022). Crucially, clinical evidence demonstrates that PK-guided IV busulfan dosing is superior to body-size dosing in patients with myeloid leukemia and myelodysplastic syndrome, yielding reduced relapse, transplant-related mortality, and overall hazard ratio (Andersson et al., 2017). Given these limitations of weight-based dosing and the demonstrated superiority of personalized approaches, therapeutic drug monitoring (TDM) is recommended as the standard of care for optimizing individual regimens (Palmer et al., 2016).

Model-informed precision dosing utilizes population PK (popPK) models combined with maximum posterior Bayesian estimation to optimize both initial and subsequent dosing regimens based on TDM measurements (Darwich et al., 2017; Shukla et al., 2020). Currently, over 40 popPK models have been developed to characterize IV busulfan PK in pediatric patients. Among the covariates, body size, age, *GST alpha 1* (*GSTA1*) genetic variations, and dosing schedule (day/time) are the most well-documented factors contributing to busulfan clearance variability (Lawson et al., 2021; Takahashi et al., 2023).

Body size descriptors, including body surface area (BSA), fat-free mass (FFM), and normal fat mass (NFM), significantly influence busulfan PK in pediatric patients (Huang et al., 2022; Lawson et al., 2021; Takahashi et al., 2023). Notably, most of these descriptors are typically incorporated into pediatric busulfan dosing individualization via allometric scaling, an approach grounded in fractal geometry principles and cross-species biological patterns (Anderson and Holford, 2008; Trame et al., 2011). Accurate quantification of allometric exponents requires data spanning the full maturation spectrum from neonates to adults (Gonzalez-Sales et al., 2022). Consequently, based on physiologically-based descriptions of body composition and theory-based allometric principles, Du et al. estimated the clearance (CL) for busulfan through allometry NFM, a maturation fraction (F_mat_), and distribution volume (V) based on FFM (Du et al., 2022).

Time-varying CL was observed over a 4-day treatment with an every-6-h dosing regimen of busulfan (Lawson et al., 2021; Takahashi et al., 2023). Specifically, CL demonstrated a progressive decline of 8.1%–20% across treatment cycles compared to baseline (Day 1), making it challenging to obtain the desired busulfan target exposure (Lawson et al., 2021). To explain this nonlinear elimination, the empirical Michaelis-Menten equation and semi-mechanistic enzyme depletion model have been employed (Langenhorst et al., 2020; Long-Boyle et al., 2015). Central to this phenomenon, busulfan-GSH conjugate serve as main intermediate metabolite, with baseline GSH levels correlating with busulfan CL; therefore, Langenhorst et al. hypothesized that busulfan-mediated GSH depletion causes nonlinear elimination (Langenhorst et al., 2020). However, the GST enzyme activity was not considered in their model.

Genetic polymorphisms in *GSTA1* are associated with 8%–27% reduction in CL (Huang et al., 2022). However, GST expression exhibits complex regulation beyond genetics, demonstrating age- and sex-dependent variations (Hines, 2008; Miyagi et al., 2009; Ten Brink et al., 2013). During pediatric development, age modulates hepatic enzyme maturation, serum protein concentrations, and body composition (water-to-fat ratio), while weight correlates with somatic growth and governs hepatic blood flow dynamics. These parameters jointly determine the evolving liver-to-body mass ratio, a critical determinant of drug metabolism capacity. GST activity decreases from infancy to early adolescence, and developmental differences in activity can markedly alter drug disposition (Gibbs et al., 1999; Hall et al., 1999).

Given this intricate interplay of physiological and pharmacological factors, comprehensive understanding of busulfan PK characteristics becomes essential for target exposure attainment. To address this, our study employs a semi-mechanistic popPK model to quantify sources of PK variability in IV busulfan exposure among pediatric HCT recipients, and mechanistically characterize GST-mediated metabolic pathways influencing drug disposition. In addition, virtual clinical trials were conducted to compare different dosing strategies, and a model-based optimal dosing regimen was recommended.

## 2 Materials and methods

### 2.1 Patients and data collection

Data were prospectively collected from 65 pediatric HCT recipients who underwent bone marrow transplantation after receiving busulfan IV during preparative chemotherapy at the Children’s Hospital of Fudan University. All patients were administered 0.8–1.2 mg/kg of busulfan via 2 h IV infusion every 6 h, depending on the patient’s WT. Patients with normal organ function were included, and those with unavailable busulfan PK data owning to difficulties in blood sampling were excluded. Demographic and pathophysiological data were prospectively obtained during routine clinical visits between August 2020 and November 2021. This study was approved by the Ethics Committee of the Children’s Hospital (ethics approval number: 2020-271) and conducted in accordance with the Declaration of Helsinki. Notably, all patients and their parents provided written informed consent to participate prior to enrolment in the study.

**FIGURE 1:**
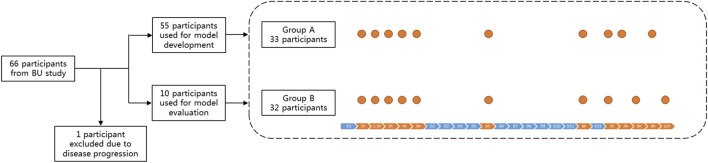
Diagram showing the prospective study dataset and trial design.

Overall, 636 whole-blood busulfan concentrations were available for model analysis. All patients received 12 doses of IV busulfan. Samples were obtained 2, 2.5, 3, 4, and 6 h following the infusion of dose 1, and pre-dose concentrations (C_0_) were collected before doses 6 and 12. Additionally, to balance the blood capacity taken and the sampling of terminal elimination, samples were collected at 2 h, 4 h, 8 h (Group A, 33 patients) and 2 h, 6 h, 12 h (Group B, 32 patients) after the infusion of dose12 (Figure 1). Furthermore, 1 mL of whole blood was collected in EDTA tubes for each sample, and all samples were stored at −80°C until analysis.

### 2.2 Determination of busulfan concentration and GST enzyme activity

Quantification of busulfan plasma concentrations was performed by a validated liquid chromatography/mass spectrometry. The assay demonstrated linearity across the analytical range of 10–5,000 ng/mL, with a lower limit of detection of 10 ng/mL. Additionally, blood samples were collected for the first time using micro-quartz colorimetry to determine GST enzyme activity. GST can catalyze the binding of GSH to 1-chlorom-2,4-ditrobenzene, which can be detected at a wavelength of 340 nm. Furthermore, 20 μL serum was mixed with the detection reagent and detected twice, before and after a 5 min water bath.

### 2.3 Semi-mechanistic population pharmacokinetic modeling

The popPK model was established using a nonlinear mixed-effects modeling approach implemented in NONMEM® (version 7.4; ICON Development Solutions, Ellicott City, MD, United States), with Pirana 2.9 serving as the interface for Perl Speaks NONMEM (PsN; version 4.9.0) to streamline model diagnostics and bootstrapping (Keizer et al., 2013). Graphical analyses were conducted through R software (version 3.5.0; http://www.r-project.org/). The first-order conditional estimation method, including η-ε interactions (FOCE-I), was employed throughout the method-building procedure (Beal et al., 1989).

The busulfan PK profile was best characterized by a two-compartment structural model with first-order elimination kinetics. Primary estimated parameters included CL, central volume of distribution (V_c_), inter-compartmental clearance (Q), and peripheral volume of distribution (V_p_). Variability components were systematically quantified through between-subject variability (BSV), inter-occasion variability (IOV), and residual unexplained variability (RUV). BSV modeled via log-normal distributions for all parameters, except Q. However, IOV was assumed to be the same across dosing occasions (Karlsson and Sheiner, 1993).

Demographic and disease-specific pathophysiological indices, and concomitant medications (Table 1) were systematically screened for potential covariates. Body size is the most identified covariate in busulfan PK modeling; therefore, four body size metrics [WT, BSA, FFM, and NFM (Text S1)] were used to determine the most suitable body size descriptor (Lawson et al., 2021). The variabilities in CL and V were characterized allometrically using body size and composition (Anderson and Holford, 2009; West et al., 1997), while busulfan metabolism maturation upon CL was evaluated using an empirical sigmoid function (F_mat_, Equation 1) (Gonzalez-Sales et al., 2022). Post-menstrual age (PMA) was a composite developmental biomarker integrating both gestational age and post-natal age.

$$F_{mat}=\left(\frac{{\;}_{\;1\;}}{\;1+{\left(\frac{PMA}{{TM}_{50}}\right)}^{-\text{Hill}}}\right)$$ (1)

where TM50 is the PMA at which maturation achieving 50% of the adult value, and Hill defines the steepness of the sigmoid decline.

**TABLE 1:** Patients demographics used to develop and evaluate population pharmacokinetic model.

Characteristics	Model development	Model evaluation
	Number or median (Range)	Number or median (Range)
No. of patients (Male/Female)^a^	55 (40/15)	10 (7/3)
No. of samples^b^	536	100
Age (years)	1.4 (0.2–14.1)	2.2 (0.5–12.7)
Weight (kg)	9.9 (2.9–29.5)	12.3 (7.5–30.0)
Height (cm)	76.0 (52.0–147.0)	85.0 (62.0–138.0)
Body mass index (kg m^-2^)	16.2 (8.1–20.9)	16.7 (14.9–19.5)
Body surface area (m^2^)	0.45 (0.22–1.09)	0.55 (0.36–1.07)
Fat-free mass (kg)	8.5 (2.5–28.0)	11.4 (6.4–23.2)
Busulfan dose (mg)	11.4 (3.0–27.6)	14.4 (6.6–28.2)
Hematocrit (%)	33.4 (23.1–42.4)	33.2 (28.2–39.4)
Total Bilirubin (μmol L^-1^)	4.2 (2.0–15.3)	3.7 (1.2–6.4)
Aspartate transferase (U L^-1^)	38.9 (16.4–344.6)	33.2 (13.2–149.1)
Albumin (g L^-1^)	38.5 (21.8–44.5)	38.0 (32.0–46.6)
Glomerular filtration rate (μmol L^-1^)	90.7 (49.7–140.5)	90.8 (65.2–132.8)
Glutathione S-transferase enzyme activity (nmol min^-1^ mL^-1^)	9.2 (0.9–20.7)	6.4 (3.7–12.4)

^a^ Data are expressed as number of patients.

^b^ Data are expressed as number of samples.

Covariate selection was conducted through a stepwise approach (Beal et al., 1989). The influence of continuous covariates was evaluated through linear, exponential, and power function models. For categorical variables (e.g., concomitant medications), intergroup comparisons were performed by analyzing fractional change differences. The variability between dosing regimen cycles in the time-dependent CL of busulfan was estimated using a linear function model and IOV on CL.

**FIGURE 2:**
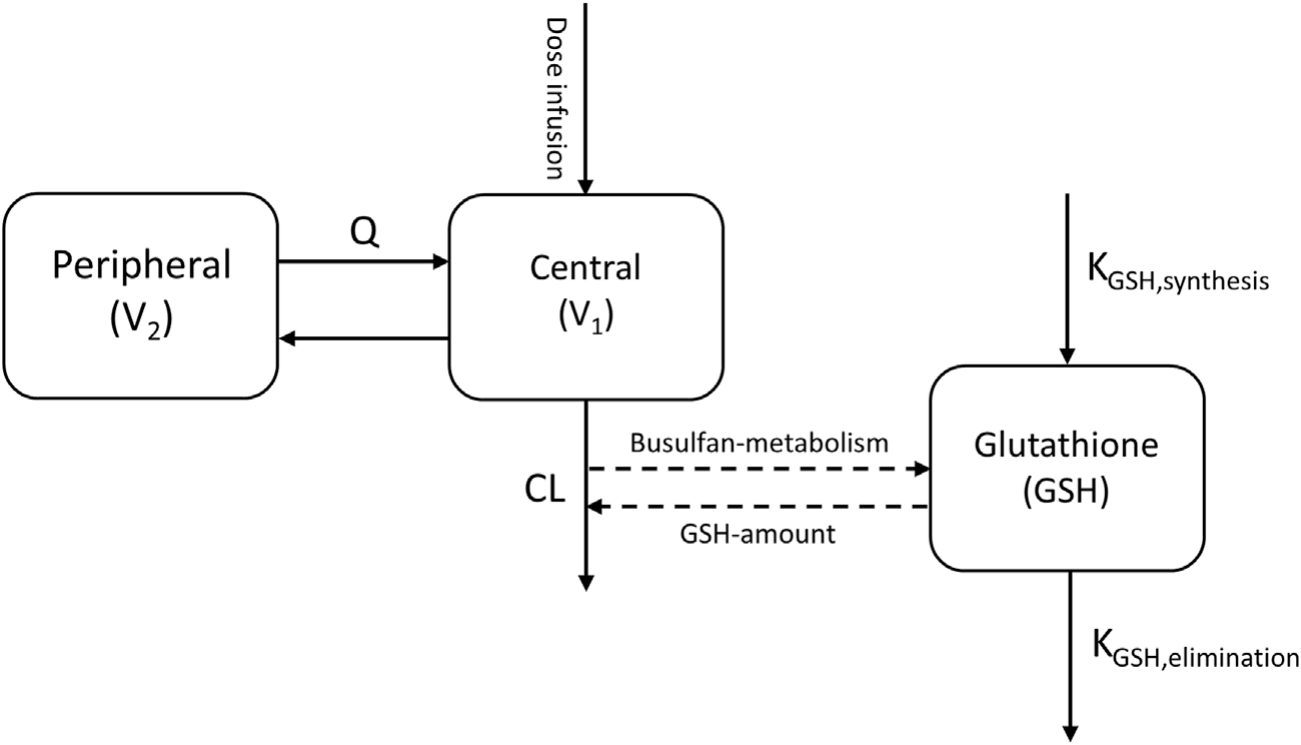
Busulfan semi-mechanistic population pharmacokinetic model structure. CL, clearance; Q, inter-compartmental clearance; V_1_, central compartment; V_2_, peripheral compartment. Dashed lines indicate the conjugation of busulfan metabolism and glutathione.

Therefore, to investigate the influence of GST enzyme activity on busulfan metabolism, the empirical model developed was used as a base, with a dedicated compartment incorporated to dynamically quantify the relative amount of GSH available over time, based on theoretical mechanisms, as reported by Langenhorst et al. (2020) (Figure 2).

The GSH compartment was initialized with a baseline normalized value of 1, and the zero-order synthesis rate was constrained to equal the first-order elimination rate constant at equilibrium, ensuring mass balance. Busulfan metabolism was modeled as a GSH-dependent conjugation process, with the scaling parameter S_GSH_ quantifying the proportionality between busulfan metabolism and corresponding GSH depletion (Equation 2).

$$\frac{{dA}_{GSH}}{dt}=\frac{S_{GSH}}{V_{1}}\times A_{GSH}\times k_{10}\times A_{{bu}_{1}}$$ (2)

Where $A_{{bu}_{1}}$ represents the amount of busulfan in the central compartment, $A_{GSH}$ represents the amount of GSH in the theoretical compartment, $V_{1}$ represents the central volume of distribution, and $k_{10}$ represents the busulfan elimination constant.

Subsequently, GST enzyme activity was tested as a continuous covariate on S_GSH_.

The visual model fit was evaluated using standard goodness-of-fit (GOF) criteria, reductions in the objective function value (OFV) for nested models, Akaike information criteria (AIC) and Bayesian information criteria (BIC) for non-nested models, and acceptable precision of estimates (Beal et al., 1989; Donohue et al., 2011). Models with lower AIC and BIC values were considered superior. A covariate was considered significant if its inclusion decreased the OFV by > 3.84 (χ^2^-test, *p* < 0.05, *df* = 1) and if backward elimination of the covariate increased the OFV by > 10.83 (χ^2^-test, *p* < 0.001, *df* = 1). Moreover, covariates were included only if they had a clear pharmacological or biological basis. During the model development process, condition numbers were calculated and maintained at ≤ 1,000 to avoid over-parameterization (Owen and Fiedler-Kelly, 2014).

In addition to GOF plots, model adequacy was rigorously evaluated through prediction-corrected visual predictive checks (pcVPCs), employing 2,000 Monte Carlo simulations to account for parameter uncertainty (Bergstrand et al., 2011). Statistical agreement was assessed by comparing the 95% confidence intervals (CIs) of simulated trajectories (median, 5th and 95th percentiles) against observed data distributions across automatically determined time intervals. Quantitative validation included visual inspection of percentile superimposition and evaluation of CI envelope coverage to confirm model robustness.

To evaluate parameter estimate robustness and precision, a nonparametric bootstrap analysis was conducted (Ette et al., 2003). Using Perl modules, 500 resampled datasets were generated through random sampling with replacement (Ette, 1997). Empirical 95% CIs and median values of the bootstrap-derived parameters with successful convergence were compared with the final model parameter estimates.

### 2.4 Virtual clinical trial of dosing strategies

Busulfan exposure is associated with both survival and toxicity in HCT recipients. Therefore, optimizing the target for busulfan cumulative exposure following all doses (cAUC) of 78–101 mg h/L during myeloablative conditioning can have a significant effect on survival chances (Bartelink et al., 2016; Lawson et al., 2021). Furthermore, to reduce the risk of sinusoidal obstructive syndrome (SOS), the maximum busulfan concentration (C_max_) should be <1.88 ng/mL (Lawson et al., 2021; Philippe et al., 2019). Therefore, Monte Carlo simulations were performed using parameter estimates from the established semi-mechanistic model, while the probabilities of target attainment for different dosing strategies were compared. Individuals involved in the evaluation dataset were regarded as a virtual population in this simulation clinical trial.

First, prediction-based metrics (median prediction error [MDPE], median absolute prediction error [MAPE], and percentage of |PE|% within 20% [F_20_] and 30% [F_30_]) were calculated to assess the model predictability (Mao et al., 2018). Second, the time-concentration profiles were simulated 200 times for each virtual individual. Busulfan doses were subsequently administered as a 2-h infusion every 6 h for 4 days (total: 16 doses). For WT-based dosing strategy, patients weighing <9 kg, 9–16 kg, 16–23 kg, 23–34 kg, and >34 kg received busulfan doses of 1 mg/kg, 1.2 mg/kg, 1.1 mg/kg, 0.95 mg/kg, and 0.8 mg/kg, respectively; however, for age-based dosing strategy, patients aged <4 years received 1 mg/kg, and those aged ≥4 years received 0.8 mg/kg (Gurlek Gokcebay et al., 2015; Nguyen et al., 2004). The cAUC was calculated using numerical integration (Bartelink et al., 2016), while the probabilities of target attainment for the two dosing strategies were compared. Finally, the busulfan dose was simulated at 0.8–1.2 mg/kg, with a step of 0.05 mg/kg for each virtual individual; subsequently, a model-based optimal dosing regimen was recommended for each involved individual.

## 3 Results

### 3.1 Patients

The demographic characteristics and clinical data of the study population are presented in Table 1. In total, 636 busulfan whole-blood samples were obtained from 65 HCT recipients. Notably, all participants were randomly divided into two groups, and 100 samples from 10 HCT recipients were used for model evaluation. Concentrations below the lower quantification limit were not included in the analysis. The median of patient postnatal age was 1.5 years (range, 0.2–14.1), with 21 patients aged <1 year. A correlation chart of patient characteristics is presented in Supplementary Figure S1.

### 3.2 Semi-mechanistic population pharmacokinetic model development

A two-compartment model with first-order absorption was selected as the base model to describe busulfan PK. The model combining proportional and additive models provided the best results for the RUV. The BSV of the mean CL/*F* in the base model was 60.8% with a relative standard error of 8.0%. The parameter estimates and associated precisions are listed in Supplementary Table S1.

Mechanistic plausibility was considered as a potential covariate, and incorporated into the base model. First, four different body size metric-based allometric candidate models were tested to determine the most suitable body size descriptor. As shown in Supplementary Table S1, the influence of patient body size on busulfan disposition was best described by allometric scaling based on NFM, with the AIC reduced by −228.1. Second, eight different models based on the NFM were compared (Supplementary Table S2), as proposed by Du et al. (2022). The AIC value of Model Ⅲ, which included NFM allometrically and busulfan metabolism maturation upon CL based on PMA physiologically, was the lowest. Therefore, Model Ⅲ was selected as the basic structural model for further analysis. Furthermore, the stepwise approach was used to screen potential covariates (Supplementary Table S3), and concomitant with fludarabine was incorporated with OFV reduced by −10.8 (*p* < 0.001).

**FIGURE 3:**
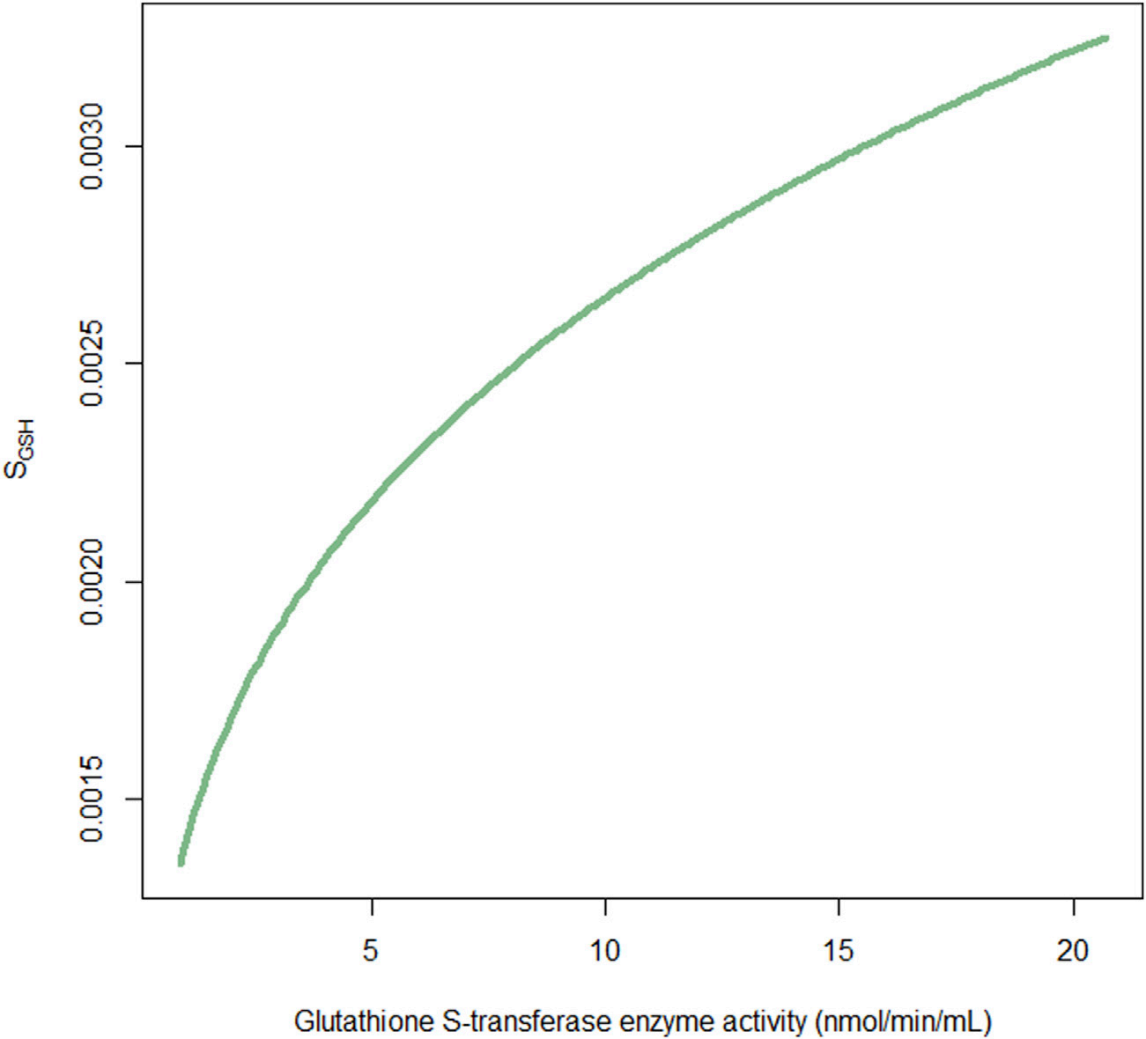
Fraction of the factor S_GSH_ *versus* glutathione S-transferase enzyme activity. S_GSH_ is used to scale the association of busulfan metabolism with relevant glutathione depletion.

The AIC value was not reduced; however, the GSH compartment was added to describe the relative amount of GSH available at any time, based on theoretical mechanisms. The S_GSH_ was fixed at 0.026 h/mg following the study by Langenhorst et al. (2020), which indicates a net relevant GSH reduction of 0.26% per hour for each milligram of busulfan metabolism scaled to a 1L central volume of distribution. The influence of GST activity on S_GSH_ was also determined exponentially. The S_GSH_ was increased by 40.6% as GST enzyme activity increased from 0.9 nmol/min/mL to 20.7 nmol/min/mL (Figure 3). The IIV of the GST slope on the S_GSH_ was 103.9%, possibly owing to the small sample size.

The additional estimation of IOV for CL significantly improved model predictions (ΔOFV −43.3, *p* < 0.001), when considering four distinct sampling occasions. During the backward process, when concomitant with fludarabine was removed from the model, the OFV increased by 5.4, which was <10.83. Therefore, this factor was excluded from the final model.

In the final model, all retained covariates significantly increased the OFV upon removal. Therefore, this model was accepted as the definitive final model. The final model parameter estimates and associated precisions are presented in Table 2. The condition number of the final model was 396.3. Shrinkage analysis for CL revealed a mean η-CL shrinkage and ε-shrinkage of 3.3% and 21.7%, respectively, which was accepted as it was below the critical threshold of 30% (Savic and Karlsson, 2009).

**TABLE 2:** Parameter estimates for the final model and the bootstrap procedure.

Parameters	Final model			Bootstrap of final model	
	Estimate	RSE (%)	Shrinkage (%)	Median	95% CI
OFV	5,678.9	—	—	—	—
AIC	5,714.9	—	—	—	—
BIC	5,792.0	—	—	—	—
CL (L h^-1^)	9.57	10.8	—	9.55	8.26–24.27
V_c_ (L)	28.2	12.2	—	27.7	22.12–31.61
Q (L h^-1^)	8.16	32.8	—	8.27	6.18–13.87
V_p_ (L)	16.1	10.7	—	16.5	14.3–20.0
Ffat_CL	0.905	50.3	—	0.943	0.189–2.144
Ffat_V_c_	0.687	28.1	—	0.688	0.298–1.078
TM50 (weeks)	45.0	23.1	—	49.6	27.8–1591.2
Hill	1.11	41.6	—	1.19	0.32–2.63
S_GSH_ (h mg^-1^)	0.00259 (fixed)	—	—	0.00259	—
GST effect on S_GSH_	0.28	103.9	—	0.27	0.017–1.14
Between-subject variability					
CL (%)	23.2	9.7	3.3	22.1	17.6–26.6
V_c_ (%)	15.6	23.4	30.6	15.9	6.1–23.8
V_p_ (%)	40.0	53.8	21.0	39.7	12.0–72.7
Inter-occasion variability					
IOV on CL	10.7	14.0	27.9	10.7	7.1–13.4
Residual variability					
Proportional (%)	11.1	10.0	21.7	10.9	8.6–13.1
Additional (mg L^-1^)	16.6	26.4	21.7	16.6	8.8–53.1

AIC, akaike information criteria; BIC, bayesian information criteria; CI, percentile confidence intervals; CL, clearance; Ffat_CL, the fraction of the fat mass for CL; Ffat_V_c_, the fraction of the fat mass for V_c_; GST, glutathione S-transferase; IOV, inter-occasion variability; OFV, objective function value; Q, inter-compartmental clearance; RSE, relative standard error; S_GSH_, the factor used to scale the relationship between busulfan metabolism and relevant glutathione depletion; TM50, the post-menstrual age at which maturation is 50% of the adult value; V_c_, central volume of distribution; V_p_, peripheral volume of distribution.

### 3.3 Model evaluation

**FIGURE 4:**
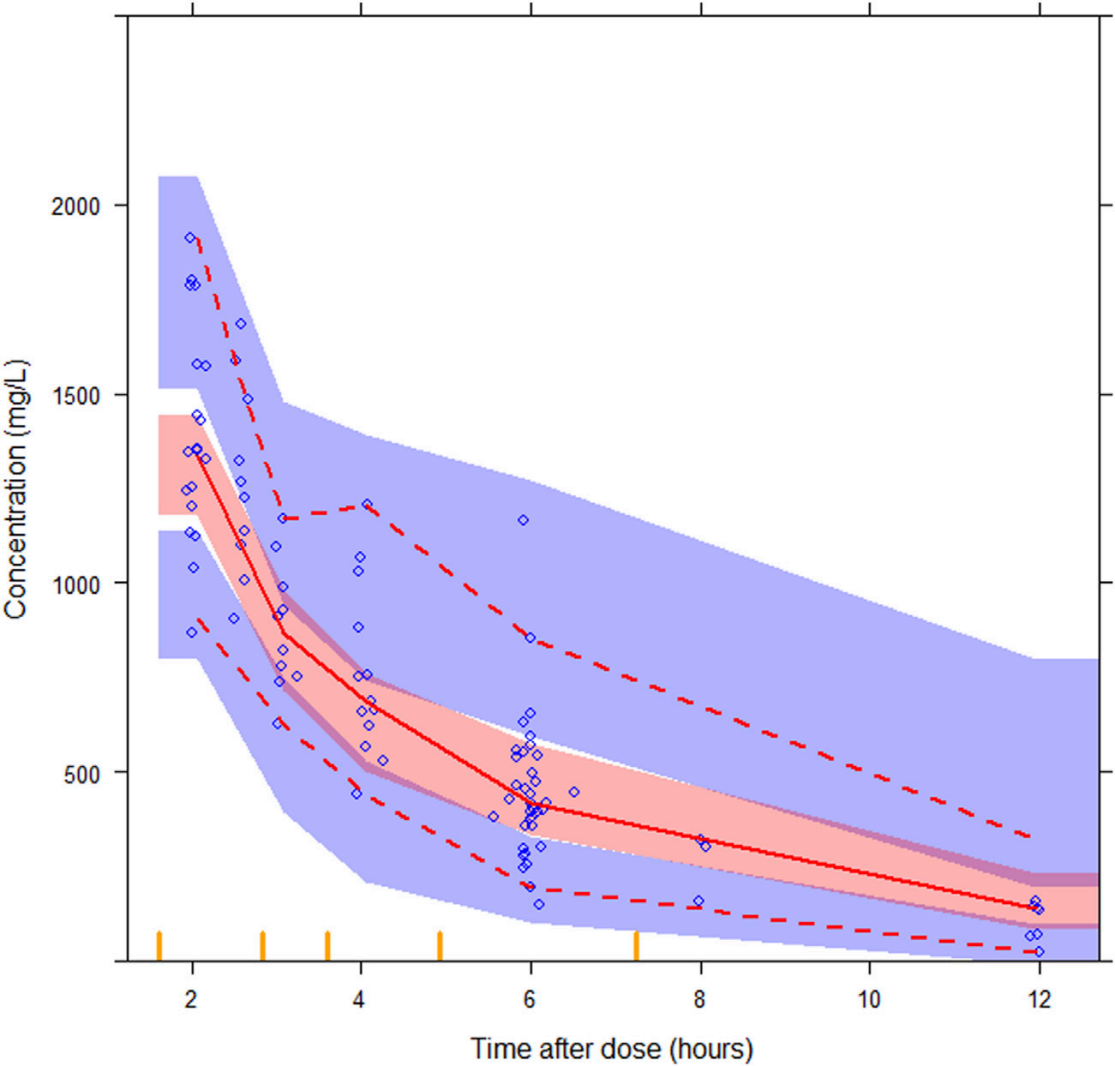
Prediction-corrected visual predictive checks (pcVPCs) for the final semi-mechanistic model. The median observed values per bin (red solid line), the 5^th^ and 95^th^ percentiles (red dashed lines) of the observations (blue circles) with the 95% confidence interval of the 5^th^ and 95^th^ percentiles (blue areas), and the confidence interval of the median (red area), are shown.

Ten patients in the evaluation group were included in the analysis to examine the predictability of the final model. The GOF plots for the final models, as presented in Supplementary Figure S2, show no apparent bias, with over 99.0% of observations falling within the four conditional weighted residuals. The pcVPC results showed good predictability of drug concentrations, as presented in Figure 4. The simulated data closely aligned with the observed data, indicating a lack of significant model misspecifications. The final model parameters were within the 95% confidence intervals of the bootstrap estimates, confirming the model’s stability (Table 2).

### 3.4 Virtual clinical trial of dosing strategies

**FIGURE 5:**
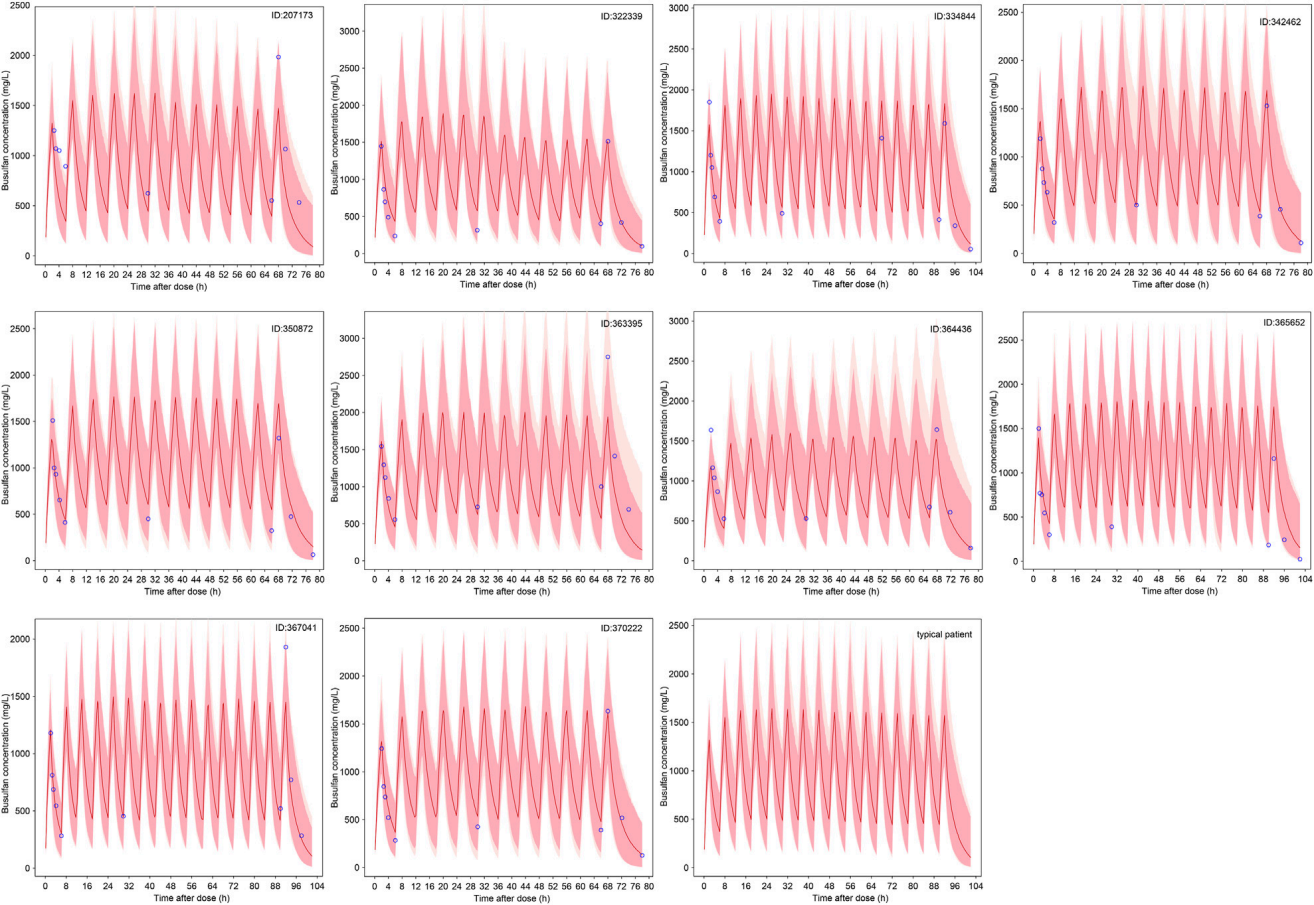
The predicted time course of busulfan concentrations in the ten individuals involved in the evaluation dataset and typical patient. The 5^th^ - 95^th^ percentiles (deep pink area) and outside 5^th^ - 95^th^ percentiles (light pink area), the median (red solid line) of the simulated data, and the observations (blue circles) are shown.

The predicted time course of busulfan concentrations in the ten individuals involved in the evaluation dataset, which was simulated based on 200 hypothetical individuals, is presented in Figure 5. All observed concentrations were within the 5th and 95th percentiles of the simulation data, showing no trends or biases. The MDPE, MAPE, F_20_, and F_30_ values were −0.44%, 16.6%, 60.0%, and 74.0%, respectively. The relatively low MDPE and MAPE values further confirmed the high prediction accuracy of the final model.

The results of the probability of target attainment for different dosing strategies based on Monte Carlo simulations are presented in Table 3. Notably, three virtual patients have 8.0%–10.5% probability of exceeding the C_max_ safety threshold of 1.88 ng/mL when receiving a WT-based dosing strategy. In contrast, this safety risk was eliminated under age-based dosing. The WT of these three patients was within 9–16 kg, indicating the risk of SOS if the patients in this group received a WT-based dosing strategy. Compared with the WT-based dosing strategy, the dosage was relatively low in patients receiving the age-based dosing strategy. No probability of concentrations >1.88 ng/mL was observed according to the Monte Carlo simulation results; however, the infant dosage was relatively high compared with the recommended optimal dosing regimen. Therefore, GST-based dosing strategy targeting cumulative cAUC was suggested, and optimal dosing regimen was recommended for typical patients (Table 3; Figure 5).

**TABLE 3:** The probability of target attainment of different dosing strategies based on Monte Carlo simulation.

ID	The characteristics of virtual population					WT-based				AGE-based				Optimal dosing regimen			
	Age (year)	WT (kg)	GST (nmol/min/mL)	GAGE (week)	FFM (kg)	Dosing regimen (mg/kg)	PTA of cAUC	Median (95% CI)	PTA of C_max_	Dosing regimen (mg/kg)	PTA of cAUC	Median (95% CI)	PTA of C_max_	Dosing regimen (mg/kg)	PTA of cAUC	Median (95% CI)	PTA of C_max_
207173	12.7	24.5	7.36	39.1	18.1	0.95	38.5%	84.6 (57.1–130.2)	100%	0.8	29.5%	71.7 (48.5–110.6)	100%	1	38.5%	88.8 (60.0–136.9)	100%
322339	2.24	12.3	10.12	38.3	11.36	1.2	35%	104.8 (70.7–161.8)	92%	1	39%	88.1 (59.5–135.5)	100%	1.05	39%	92.3 (62.3–142.1)	100%
334844	1.67	10.5	9.2	39.1	9.35	1.2	35.5%	106.4 (71.8–164.0)	91%	1	39.5%	89.4 (60.4–137.5)	100%	1.05	40%	93.7 (63.6–144.4)	100%
342462	7.74	23.6	6.44	38.1	17.63	0.8	32%	73.2 (49.5–113.0)	100%	0.95	39%	86.4 (58.4–133.0)	100%	1	40%	90.7 (61.3–139.6)	100%
350872	0.92	7.5	6.44	39	6.76	1	40%	92.6 (62.7–142.6)	100%	1	40%	92.6 (62.7–142.6)	100%	1.05	40.5%	97.1 (65.6–149.3)	100%
363395	2.28	13	12.42	37.6	11.4	1.2	35%	106.0 (71.5–163.8)	89.5%	1	40%	89.2 (60.2–137.3)	100%	1.05	40.5%	93.4 (63.0–144.0)	100%
364436	0.46	7.5	3.68	38.6	6.38	1	35%	106.6 (72.3–164.4)	100%	1	35%	106.6 (72.3–164.4)	100%	0.9	40.5%	96.3 (65.3–148.7)	100%
365652	0.6	7.5	3.68	38.6	6.69	1	37.5%	101.3 (68.7–156.3)	100%	1	37.5%	101.3 (68.7–156.3)	100%	0.9	40.5%	91.5 (62.0–141.4)	100%
367041	11.16	24.9	4.14	37.1	23.17	0.95	39%	84.9 (57.5–131.1)	100%	0.8	30%	71.9 (48.7–111.2)	100%	1.05	40%	93.5 (63.3–144.1)	100%
370222	9.33	30	11.04	38.0	22.0	0.95	40%	90.0 (60.8–138.4)	100%	0.8	34.5%	76.3 (51.6–117.5)	100%	0.95	40%	90.0 (60.8–138.4)	100%
typical patient	1.4	9.9	10.12	38.3	8.8	1.2	32.5%	108.4 (73.1–167.1)	91%	1	40.5%	91.1 (61.6–140.0)	100%	1	40.5%	91.1 (61.6–140.0)	100%

cAUC, cumulative exposure following all doses; CI, confidence interval; C_max_, the maximum concentration; FFM, fat-free mass; GAGE, gestational age; GST, glutathione S-transferases; PTA, probability of target attainment; WT, weight.

## 4 Discussion

Over 40 busulfan popPK studies have been reported, of which 68% were developed predominantly in children, from which 69% and 26% of models were developed using first-order elimination and time-varying CL, respectively (Takahashi et al., 2023). Thus far, only one study has been based on a semi-mechanistic enzyme depletion model (Langenhorst et al., 2020). Furthermore, the effects of GST activity remain uncharacterized. Therefore, in this prospective study, factors affecting the PK variability of IV busulfan in pediatric HCT recipients were identified, and the effect of GST activity on the time-varying CL of busulfan was investigated using a semi-mechanistic popPK model.

In the final model, busulfan disposition was best described by including NFM allometrically as a body size metric and age-dependent maturation function. The influence of GST activity on busulfan metabolism was added exponentially based on theoretical mechanisms. The IOV was observed between dosing occasions. The developed model described busulfan PK IIV well, and model-based target attainment of different dosing strategies was evaluated.

Body size scalers and age factors were the most commonly identified covariates impacting busulfan PK in pediatric patients (Du et al., 2022; Takahashi et al., 2023). Given the broad age range of our cohort (0.2–14.1 years), we implemented NFM, a theory-based size descriptor that divides WT into FFM and fat mass (Gonzalez-Sales et al., 2022), to quantify the effect of body size and composition on busulfan PK, consistent with the study by McCune et al. (2014), Van Hoogdalem et al. (2020). In the final model, the fat mass fraction was 90.5% for CL and 68.7% for V, which was reported as 50.9% for CL, 20.3% for V by and 69.2% for CL by Du et al. (2022), McCune et al. (2014). This inconsistency in fat mass may be caused by differences in age distributions between the studies. The mean ages in the study by McCune et al. and Du et al. were 9.8 years (0.1–65.8 years) and 6.1 years (0.6–17 years), respectively. In contrast, our population averaged 2.9 years. The estimated typical CL and V_c_ standardized to 70-kg adult patient were 9.57 L/h and 28.2 L, respectively, which is consistent with previous studies (Takahashi et al., 2023).

To characterize developmental pharmacology, age-dependent physiological maturation functions were applied to describe changes in CL with age. Consistent with established principles, CL maturation begins before birth, making PMA more physiologically relevant than postnatal age (Anderson and Holford, 2008). According to our results, the maturation of busulfan CL reached 50% of adult values at 45 weeks PMA, which is comparable with McCune et al.’s study (TM50 = 45.7) (McCune et al., 2014). Savic et al. reported that CL increases approximately 1.7-fold between 6 weeks and 2 years by adding a nonlinear function of CL *versus* age to describe CL maturation (Savic et al., 2013). McCune et al. also reported that size-standardized CL reaches 95% of adult values at 2.5 postnatal years (McCune et al., 2014). Although no information on adults was available in our study, the same tendency was observed (Supplementary Figure S3), suggesting future studies should expand sample sizes to validate these maturation dynamics.

The formation of busulfan-GSH conjugates mechanistically depends on both GSH availability and depletion kinetics. Consistent with this, baseline GSH concentrations and GST polymorphisms are associated with busulfan CL (Almog et al., 2011; Takahashi et al., 2023). Among the five GST classes, the *GSTA1* haplotype’ impact on CL exhibits age-dependency, reflecting the protein abundance of GSTAs in the liver increases after birth to reach adult levels during infancy (Strange et al., 1985; Strange et al., 1989). The maturation of liver drug enzymes may partially contribute to the early age-dependent PK of busulfan, indicating that GST enzyme activity may be more suitable than GST polymorphisms when assessing the influence of GST enzymes on busulfan disposition. To test this hypothesis, we incorporated GST activity effects into a semi-mechanistic PK model. Theoretically, increased GST activity would have faster GSH depletion, resulting in higher CL. However, further information on the complete developmental profile of GST enzyme activity is required in future studies.

Apart from the intra-individual variability induced by physiological maturation, busulfan-mediated GSH depletion during the treatment process may result in time-varying CL of busulfan (Langenhorst et al., 2020). The initial metabolism of busulfan occurs primarily through conjugation with endogenous GSH (spontaneously and through GST catalysis) (Myers et al., 2017). Consequently, the depletion of whole-blood GSH may contribute to the observed metabolism-dependent CL reduction (Almog et al., 2011), with clinical studies documenting 17% average CL decline from treatment initiation to day 3 (Schreib et al., 2023). Notably, no specific tendencies were noted in our study; however, IOV was included in the random-effects model to estimate course-to-course variability. The complex relationship between hepatic and blood GSH concentrations during dynamic changes may further contribute to IOV.

Other potential covariates, such as concomitant medications, disease type, and other pathophysiological indicators, were also investigated (Supplementary Table S3); however, they showed no significant effect on the busulfan PK process in this study. Notably, the effect of fludarabine co-administration and disease type has been controversial between studies (Almog et al., 2011; McCune et al., 2014; Takahashi et al., 2023). Furthermore, drug-drug interactions associated with GST depletion, such as N-acetylcysteine, should be used cautiously in the clinic (Palmer et al., 2016; Schreib et al., 2023).

Current busulfan dosing in pediatric HCT patients is based on the recommendations of regulatory agencies, such as the European Medicines Agency and U.S. Food and Drug Administration (Gurlek Gokcebay et al., 2015). Our virtual trial results corroborate previous clinical findings that WT-based dosing strategy has a higher risk of SOS compared with age-based dosing strategy (Gurlek Gokcebay et al., 2015). Specifically, Gurlek et al. previously reported that WT-based dosing was a predictor of SOS (Hazard ratio: 9.46, *p* = 0.009), with an SOS of 42% compared with the 5% for those receiving age-based dosing (Gurlek Gokcebay et al., 2015). The incidence of SOS is reported at 16% (range: 0%–34%), with risk factors including combining busulfan with cyclophosphamide, *GSTA1* genotypes, age, weight <9 kg, weight-based dosing, and the use of once-daily IV busulfan (Lawson et al., 2021). Therefore, model-based precision dosing based on busulfan cAUC should be performed to optimize the dose regimen.

One potential limitation of this study was the lack of assessment of active GSH levels and relationship between plasma and liver GSH levels during GSH resynthesis. Therefore, full GSH dynamics could not be reconstructed in current analysis. Furthermore, comprehensive developmental profiling of GST activity from neonates to adults requires larger sample sizes to quantify its impact on busulfan CL. Additionally, as a single-center study, multicenter validation is required to enhance the model predictability.

In conclusion, we developed a semi-mechanistic popPK model to investigate the PK variability of IV busulfan in pediatric HCT recipients. Our findings demonstrate that physiologically-based descriptions of body composition according to allometry NFM, F_mat_, and GST enzyme activity may mediate busulfan PK variability. Virtual clinical trial revealed that WT-based dosing strategy has a higher risk of SOS than age-based dosing strategy. Therefore, model-informed precision dosing targeting cumulative cAUC is essential for optimizing dosing regimens.

## Data Availability

The original contributions presented in the study are included in the article/Supplementary Material, further inquiries can be directed to the corresponding author.
