# Supplementary material for: Semi-mechanistic population pharmacokinetic model incorporating glutathione S-transferase activity for personalized busulfan dosing in pediatric allogeneic hematopoietic cell transplantation
Source: Front Pharmacol. 2025 Aug 29;16:1632588. doi: 10.3389/fphar.2025.1632588 (PMC12426406; doi:10.3389/fphar.2025.1632588)
Supplement: Supplementary file 1 [file Supplementaryfile1.docx]

# Supporting information

Additional Supporting Information may be found in the online version of this article at the publisher’s web-site:

***Text S1*** The model used to predict four body size metrics

***Table S1*** Parameter estimates for the base model with and without body size metrics

***Table S2*** Parameter estimates of the eight NFM-dependent clearance candidate models

***Table S3*** Covariates screen process based on the basic structural model

***Text S2*** NONMEM code for final model including covariates

***Figure S1*** The correlation chart of the patient characteristics. Scatter plots with locally weighted scatterplot smoothing (LOESS) trend lines (red lines) are shown in lower left panels. Density distributions for each variables are shown in diagonal panels. Pearson correlation coefficients are shown in upper right panels. ALB, albumin; ALP, alkaline phosphatase; ALT, alanine transaminase; AST, aspartate aminotransferase; BMI, body mass index; BSA, body surface area; FFM, fat-free mass; GAGE, gestational age; GEND, gender; GST, glutathione S-transferase; GLB, globulin; HB, hemoglobin; HCT, hematocrit; HT, height; PLT, platelet count; RBC, red blood cell count; TBIL, total bilirubin; WBC, white blood cell count; WT, body weight

***Figure S2*** Diagnostic goodness-of-fit plots for the final semi-mechanistic model. (a) observations versus population predictions; (b) observations versus individual predictions; (c) conditional weighted residuals (CWRES) versus population predictions; (d) CWRES versus time after dose. (a - d) The locally weighted regression line (red dashed lines). (a, b) the line of unity (black solid lines), and (c, d) y = 0 (solid lines) are shown.

***Figure S3*** Empirical Bayes estimates of busulfan clearance versus postnatal age. The model-predicted typical clearance (red dashed line) and the individual clearance (blue circles) are shown.
