# Supplementary material for: Semi-mechanistic population pharmacokinetic model incorporating glutathione S-transferase activity for personalized busulfan dosing in pediatric allogeneic hematopoietic cell transplantation
Source: Front Pharmacol. 2025 Aug 29;16:1632588. doi: 10.3389/fphar.2025.1632588 (PMC12426406; doi:10.3389/fphar.2025.1632588)
Supplement: Supplementary file 2 [file Supplementaryfile3.docx]

# Electronic Supplementary Material

##
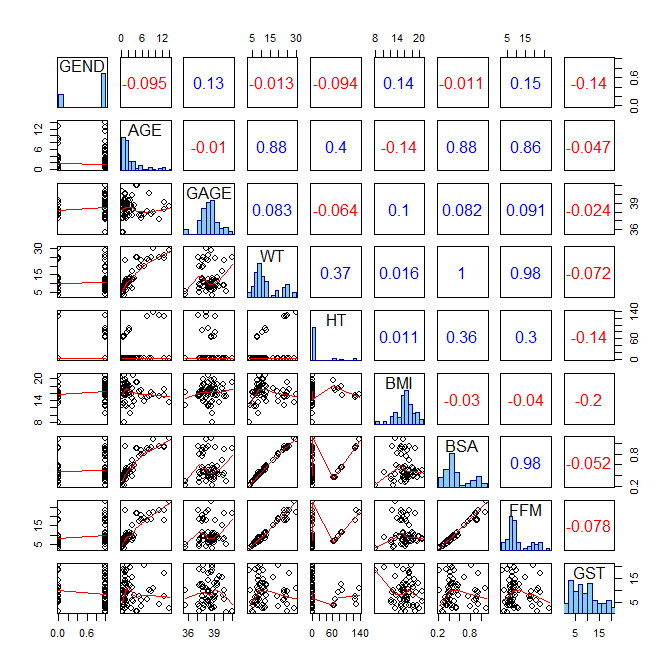
Supplementary Figure S1A

##
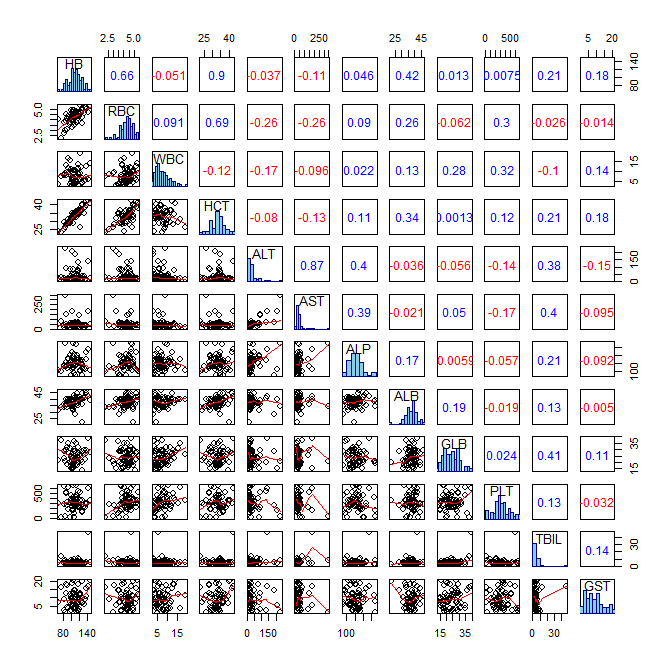
Supplementary Figure S1B

##
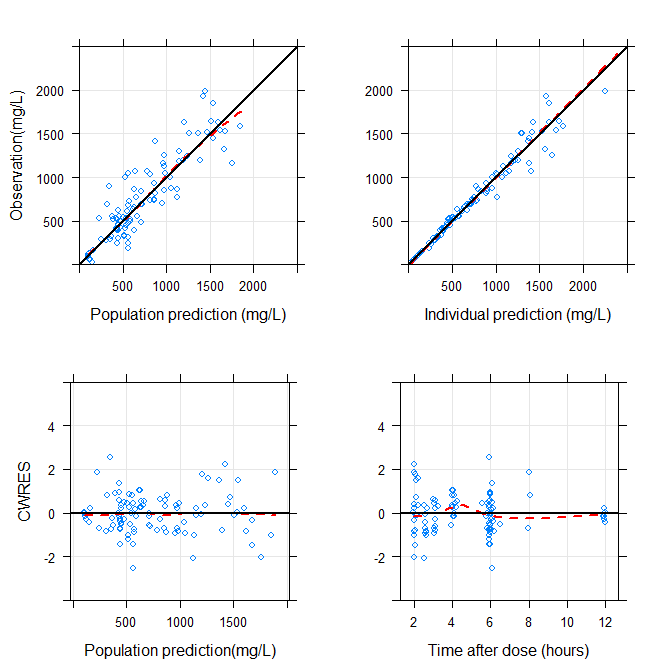
Supplementary Figure S2

d

c

b

a

##
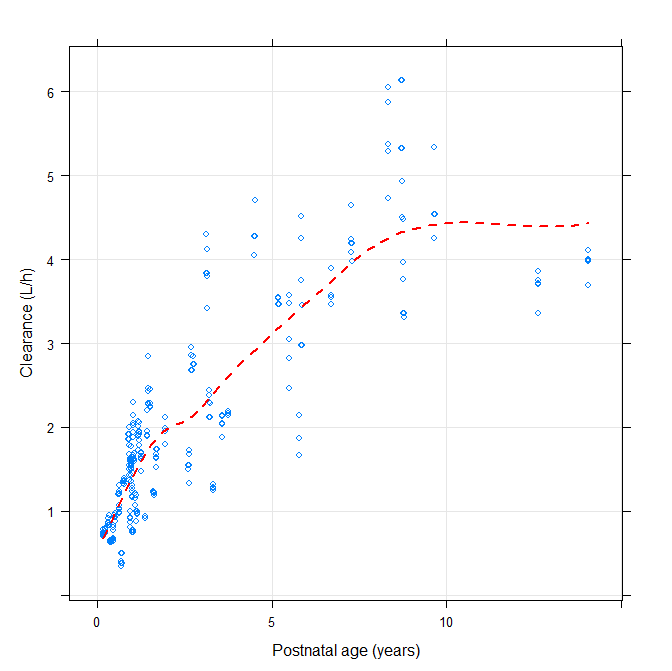
Supplementary Figure S3
