# Supplementary material for: Semi-mechanistic population pharmacokinetic model incorporating glutathione S-transferase activity for personalized busulfan dosing in pediatric allogeneic hematopoietic cell transplantation
Source: Front Pharmacol. 2025 Aug 29;16:1632588. doi: 10.3389/fphar.2025.1632588 (PMC12426406; doi:10.3389/fphar.2025.1632588)
Supplement: Supplementary file 3 [file Supplementaryfile2.docx]

# Electronic Supplementary Material

## Supplementary Text S1 The model used to predict four body size metrics

The model used to predict body surface area (BSA) as follows:

BSA (m^2^) = (HT [cm]×WT [kg]/3600)^0.5^ (1)

The semi-mechanistic model used to predict fat-free mass (FFM) as follows [[1](#_ENREF_1)]:

FFM (male) = $\frac{9270\times WT}{6680+216\times BMI}$ (2)

FFM (female) = $\frac{9270\times WT}{8780+244\times BMI}$ (3)

Where WT is measured in kilograms, HT is measured in centimeters, BMI is calculated following the formula: BMI = weight (kg)/height^2^ (m^2^), *BMI* body mass index, *HT* height, *WT* body weight

The semi-mechanistic model used to predict normal fat mass (NFM) as follows [[2](#_ENREF_2)]:

NFM = FFM + Ffat×(WT -FFM) (4)

The fraction of fat mass (Ffat) represents the fat mass (WT-FFM) equivalent to FFM in terms of allometric size

## Supplementary Table S1 Parameter estimates for the base model with and without body size metrics

| Parameters | Base model | | Base model with WT | | Base model with BSA | | Base model with FFM | | Base model with NFM | |
| --- | --- | --- | --- | --- | --- | --- | --- | --- | --- | --- |
|  | Estimate | RSE (%) | Estimate | RSE (%) | Estimate | RSE (%) | Estimate | RSE (%) | Estimate | RSE (%) |
| OFV | 5956.4 | / | 5735.7 | / | 5778.5 | / | 5738.4 | / | 5724.3 | / |
| AIC | 5974.4 | / | 5753.7 | / | 5796.5 | / | 5756.4 | / | 5746.3 | / |
| BIC | 6012.9 | / | 5798.3 | / | 5835.1 | / | 5795.0 | / | 5793.5 | / |
| CL (L h^-1^) | 1.8 | 8.5 | 1.7 | 4.0 | 1.7 | 5.0 | 1.7 | 4.2 | 7.3 | 4.2 |
| V_c_ (L) | 3.4 | 51.8 | 3.7 | 19.8 | 3.5 | 21.7 | 3.8 | 20.9 | 26.0 | 20.1 |
| Q (L h^-1^) | 3.89 | 77.6 | 2.5 | 40.4 | 2.9 | 38.8 | 2.3 | 45.7 | 10.4 | 44.6 |
| V_p_ (L) | 3.2 | 35.2 | 2.6 | 16.8 | 2.7 | 17.1 | 2.5 | 18.5 | 17.7 | 18.8 |
| Ffat_CL | / | / | / | / | / | / | / | / | 0.837 | 55.9 |
| Ffat_V_c_ | / | / | / | / | / | / | / | / | 0.704 | 31.7 |
| Between-subject variability | | | | | | | | | | |
| CL (%) | 60.8 | 8.0 | 29.1 | 7.8 | 35.2 | 8.3 | 30.4 | 7.7 | 29.2 | 7.8 |
| V_c_ (%) | 81.5 | 28.9 | 13.0 | 38.5 | 23.5 | 20.4 | 18.1 | 29.0 | 13.6 | 33.8 |
| V_p_ (%) | 59.2 | 34.0 | 37.8 | 43.0 | 41.1 | 37.3 | 39.1 | 46.7 | 38.2 | 46.9 |
| Residual variability | | | | | | | | | | |
| Proportional (%) | 13.5 | 11.2 | 13.6 | 9.0 | 13.6 | 9.7 | 13.5 | 9.4 | 13.5 | 9.1 |
| Additional (mg L^-1^) | 26.9 | 41.4 | 24.6 | 36.7 | 26.7 | 36.7 | 25.0 | 36.6 | 25.2 | 37.5 |

AIC, akaike information criteria; BIC, bayesian information criteria; CL, clearance; Ffat_CL, the fraction of the fat mass for CL; Ffat_V_c_, the fraction of the fat mass for V_c_; OFV, objective function value; Q, inter-compartmental clearance; RSE, relative standard error; V_c_, central volume of distribution; V_p_, peripheral volume of distribution

## Supplementary Table S2 Parameter estimates of the eight NFM-dependent clearance candidate models

| Parameters | Model Ⅰ | Model Ⅱ | Model Ⅲ | Model Ⅳ | Model Ⅴ | Model Ⅵ | Model Ⅶ | Model Ⅷ |
| --- | --- | --- | --- | --- | --- | --- | --- | --- |
|  | 3/4 Allometric model | Simple exponent model | 3/4 Allometric and maturation function model | Age-cutoff separated model^a^ | Age-cutoff separated model^b^ | Weight-dependent exponent model^c^ | FFM-dependent exponent model^c^ | Age-dependent exponent model^c^ |
| Model description | CL×(NFM/56.1)^3/4^ | CL×(NFM/56.1)*^k1^* | CL×(NFM/56.1)^3/4^×F*_mat_* | CL×(NFM/56.1)*^k1^* | CL×(NFM/56.1)*^k1^* | CL×(NFM/56.1)*^k1^* | CL×(NFM/56.1)*^k1^* | CL×(NFM/56.1)*^k1^* |
| OFV | 5724.2 | 5714.0 | 5703.6 | 5710.2 | 5738.1 | 5710.8 | 5707.0 | 5711.4 |
| AIC | 5746.3 | 5738.0 | 5729.6 | 5738.2 | 5766.1 | 5740.8 | 5737.0 | 5741.4 |
| BIC | 5793.5 | 5789.4 | 5785.3 | 5798.2 | 5826.1 | 5805.1 | 5801.3 | 5805.7 |
| CL (L h^-1^) | 7.33 (4.2) | 11.0 (14.6) | 9.66 (8.9) | 7.59 (25.6)/10.8 (14.1) | 7.86 (20.2)/10.4 (13.7) | 9.46 (18.1) | 8.29 (18.6) | 9.42 (22.2) |
| V_c_ (L) | 26.0 (20.1) | 27.4 (22.9) | 26.3 (22.8) | 26.6 (21.8) | 21.9 (8.9) | 27.1 (24.1) | 26.8 (21.8) | 27.7 (20.3) |
| Q (L h^-1^) | 10.4 (44.6) | 13.4 (56.3) | 10.0 (52.9) | 12.9 (52.1) | 16.6 (21.3) | 11.9 (55.3) | 10.6 (50.8) | 11.1 (60.8) |
| V_p_ (L) | 17.7 (18.8) | 16.6 (23.8) | 17.4 (21.8) | 17.3 (21.0) | 19.7 (7.0) | 16.8 (25.1) | 17.0 (22.0) | 16.4 (20.9) |
| Ffat_CL | 0.837 (55.9) | 0.962 (37.0) | 1.0 (46.3) | 0.919 (39.2) | 1.1 (40.1) | 0.908 (40.3) | 1.14 (36.6) | 1.09 (42.8) |
| Ffat_V_c_ | 0.704 (31.7) | 0.692 (33.8) | 0.688 (34.6) | 0.711 (31.5) | 0.73 (27.9) | 0.698 (33.7) | 0.703 (33.4) | 0.685 (33.4) |
| k_1_ | 0.75 | 0.963 (7.8) | 0.75 | 0.834 (13.2)/0.936 (8.8) | 0.836 (10.8)/0.84 (11.5) | / | / | / |
| k_1_=k_0_-k_max_/{[1+(Weight or FFM or Age/k_50_)^-Hill^]} | | | | | | | | |
| TM50 | / | / | 44.7 (20.6) | / | / | / | / | / |
| k_0_ | / | / | / | / | / | 0.939 (8.6) | 0.859 (10.1) | 0.889 (12.5) |
| k_max_ | / | / | / | / | / | 0.0779 (54.6) | 0.139 (27.6) | 0.183 (96.2) |
| k_50_ | / | / | / | / | / | 7.43 (576.0) | 8.49 (0.3) | 6.53 (4.2) |
| Hill | / | / | 1.26 (38.2) | / | / | 829 (3872.1) | 449 (155.5) | 79.3 (152.6) |
| CL (%) | 29.2 (7.8) | 25.6 (10.0) | 24.0 (9.2) | 25.5 (10.0) | 22.4 (9.4) | 24.7 (10.2) | 24.5 (10.7) | 25.4 (9.1) |
| V_c_ (%) | 13.6 (33.8) | 13.5 (31.3) | 13.2 (31.5) | 13.9 (31.5) | 19.2 (22.6) | 13.3 (33.2) | 12.6 (33.6) | 13.7 (29.6) |
| V_p_ (%) | 38.2 (46.9) | 41.4 (55.3) | 39.6 (52.5) | 39.2 (51.3) | 24.0 (18.8) | 40.6 (56.1) | 40.5 (49.4) | 42.8 (54.9) |
| Proportional (%) | 13.5 (9.1) | 13.5 (9.0) | 13.4 (9.4) | 13.5 (9.1) | 9.5 (13.4) | 13.6 (10.0) | 13.5 (8.9) | 13.4 (9.3) |
| Additional (mg L^-1^) | 25.2 (37.5) | 25.6 (38.6) | 26.6 (41.7) | 25.4 (39.7) | 69.0 (21.6) | 25.4 (51.5) | 25.9 (36.9) | 25.9 (40.8) |

^a^ the cutoff value is 1 year of age

^b^ the cutoff value is 2 years of age

^c^ For Model Ⅵ, Model Ⅶ and Model Ⅷ, k_1_=k_0_-k_max_/{[1+(Weight or FFM or Age/k_50_)^-Hill^]}

AIC, akaike information criteria; BIC, bayesian information criteria, CL, clearance; Ffat_CL, fat fraction for CL; Ffat_V_c_, fat fraction for V_c_; FFM, fat-free mass; Hill, hill coefficient for maturation; k_max_, the maximum decrease of the exponent; k_50_, the weight (Model Ⅵ), FFM (Model Ⅶ) or Age (Model Ⅷ) at which a 50% decrease in the maximum decrease is attained; OFV, objective function value; Q, inter-compartmental clearance; RSE, relative standard error; TM50, the post-menstrual age at which maturation is 50% of the adult value; V_c_, central volume of distribution; V_p_, peripheral volume of distribution

## Supplementary Table S3 Covariates screen process based on the basic structural model

| No. | Model description | OFV | ΔOFV |  |
| --- | --- | --- | --- | --- |
| forward process (*df* = 1, *p* < 0.05, χ^2^ = 3.84) | | | | |
| 1 | Model Ⅲ | 5703.6 | / | basic structural model |
| 2 | HCT on CL-linear | 5702.4 | -1.2 |  |
| 3 | HCT on CL-power | 5700.7 | -2.9 |  |
| 4 | HCT on CL-exponential | 5700.4 | -3.3 |  |
| 5 | TBIL on CL-linear | 5703.3 | -0.3 |  |
| 6 | TBIL on CL-power | 5703.6 | -0.1 |  |
| 7 | TBIL on CL-exponential | 5703.3 | -0.3 |  |
| 8 | ALT on CL-linear | 5702.0 | -1.7 |  |
| 9 | ALT on CL-power | 5702.2 | -1.5 |  |
| 10 | ALT on CL-exponential | 5702.0 | -1.7 |  |
| 11 | ALB on CL-linear | 5700.4 | -3.2 |  |
| 12 | ALB on CL-power | 5700.6 | -3.0 |  |
| 13 | ALB on CL-exponential | 5700.3 | -3.3 |  |
| 14 | CRP on CL-linear | 5703.6 | 0.002 |  |
| 15 | CRP on CL-power | 5703.6 | 0.002 |  |
| 16 | CRP on CL-exponential | 5703.6 | 0.002 |  |
| 17 | eGFR on CL-linear | 5702.9 | -0.75 |  |
| 18 | eGFR on CL-power | 5703.3 | -0.35 |  |
| 19 | eGFR on CL-exponential | 5702.9 | -0.71 |  |
| 20 | PID on CL-categorical | 5702.5 | -1.1 |  |
| 21 | FDLB on CL-categorical | 5692.8 | -10.8 | candidate model |

ALB, Albumin; ALT, Alanine Aminotransferase; CL, clearance; CRP, C-reactive protein; eGFR, estimated Glomerular filtration rate; FDLB, concomitant with fludarabine; HCT, hematocrit; OFV, objective function value; PID, patients diagnosed with primary immunodeficiency diseases

## Supplementary Text S2 NONMEM code for final model including covariates

$INPUT -----------------------------------------

$DATA ----------------------------------------- IGNORE=#

$SUBROUTINE ADVAN6 TOL=4

$MODEL NCOMP=3

COMP = (CENTRAL, DEFDOSE) ; Central compartment

COMP = (PERIPH) ; Peripheral compartment

COMP = (GSH) ; GSH compartment

$PK

; -------------------- MATURATION FUNCTION ESTIMATION --------------------

PMA = AGE * 365/7 + GAGE ; Post-menstrual age

TM50 = THETA(7) ; Maturation half-time

HILL = THETA(8) ; Maturation steepness

FMAT = 1/(1 + (PMA/TM50)**(-HILL)) ; the maturation function

; ---------------------------------- NFM ESTIMATION ----------------------------------

FFAT_CL = THETA(5) ; Fat fraction for clearance

FFAT_V = THETA(6) ; Fat fraction for volume

FAT = WT - FFM ; the difference of fat mass

; FFM standard size for WT=70kg HT=1.76m is set to 56.1

NFMSTD_CL = 56.1 + FFAT_CL * (70 - 56.1) ; standard size individual with NFM_std,CL_

NFMSTD_V = 56.1 + FFAT_V * (70 - 56.1) ; standard size individual with NFM_std,V_

NFMCL = FFM + FFAT_CL * FAT

FSIZCL = (NFMCL/NFMSTD_CL)**0.75 ; NFM effect on clearance

NFMV = FFM + FFAT_V * FAT

FSIZV = NFMV/NFMSTD_V ; NFM effect on volume

; ------------------------------- OCC SETTING FOR IOV -------------------------------

OCC1 = 0

IF (OCC.EQ.1) OCC1 = 1

OCC2 = 0

IF (OCC.EQ.5) OCC2 = 1

OCC3 = 0

IF (OCC.EQ.11) OCC3 = 1

OCC4 = 0

IF (OCC.EQ.12) OCC4 = 1

OCD = OCC1 * ETA(4) + OCC2 * ETA(5) + OCC3 * ETA(6) + OCC4 * ETA(7)

; ---------------------------- PK PARAMETER ESTIMATION ----------------------------

TVCL = THETA(1) * FSIZCL * FMAT ; Population clearance

CL = TVCL * EXP(ETA(1)+OCD) ; Individual clearance

TVV1 = THETA(2) * FSIZV ; Population central volume

V1 = TVV1 * EXP(ETA(2)) ; Individual central volume

Q = THETA(3) * FSIZCL ; Inter-compartment clearance

TVV2 = THETA(4) * FSIZV ; Population peripheral volume

V2 = TVV2 * EXP(ETA(3)) ; Individual peripheral volume

; ----------------- GST ACTIVITY ON BUSULFAN METABOLISM -----------------

TVS_GSH = THETA(9) ; Scaling parameter S_GSH_

SLOPE_GST = THETA (10) ; GST effect on S_GSH_

S_GSH = TVS_GSH * (GST/9.2) ** SLOPE_GST

K10 = CL/V1

K12 = Q/V1

K21 = Q/V2

KGSH = S_GSH/V1

S1 = V1 / 1000 ; Scale factor for central volume

S2 = V2 / 1000 ; Scale factor for peripheral volume

;--------------------------

; Initial conditions

A_0(3) = 1 ; The GSH compartment was initialized with a baseline normalized value of 1

;--------------------------

$DES

DADT(1) = - (K12 + K10 * A(3)) * A(1) + K21 * A(2)

DADT(2) = K12 * A(1) - K21 * A(2)

DADT(3) = KGSH * A(3) * K10 * A(1)

$ERROR

IPRED = F ; Individual prediction

IRES = DV - IPRED ; Individual residual

DEL = 0 ; Used to ignore the record DV=0

IF (DV .EQ. 0) DEL = 1

IWRES = (1-DEL) * IRES / (DV + DEL) ; Individual weighted residual

Y = F * EXP (EPS (1)) + EPS (2) ; Proportional error model

$THETA

(0, 9.57) ; Clearance

(0, 28.2) ; Central volume of distribution

(0, 8.16) ; Inter-compartment clearance

(0, 16.1) ; Peripheral volume of distribution

(0, 0.905) ; Fat fraction for clearance

(0, 0.687) ; Fat fraction for volume

(0, 45.0) ; TM50

(0, 1.11) ; Hill coefficient

(0.00259, FIXED) ; S_GSH_ was fixed at 0.026 h/mg (Langenhorst et al.)

(0, 0.28) ; GST effect on S_GSH_

$OMEGA

0.539 ; BSV of clearance

0.0244 ; BSV of central volume

0.16 ; BSV of peripheral volume

$OMEGA BLOCK (1) 0.0115

$OMEGA BLOCK (1) SAME

$OMEGA BLOCK (1) SAME

$OMEGA BLOCK (1) SAME

$SIGMA

0.0124 ; Exponential error

276 ; Additive error

$ESTIMATION METH=1 INTE MAXEVAL=9999 PRINT=10 NOABORT POSTHOC
